# Supplementary material for: mRNA-Driven Generation of Transgene-Free Neural Stem Cells from Human Urine-Derived Cells
Source: Cells. 2019 Sep 6;8(9):1043. doi: 10.3390/cells8091043 (PMC6769943; doi:10.3390/cells8091043)
Supplement: Supplementary file 1 [file cells-08-01043-s001.pdf]

Supplementary Materials

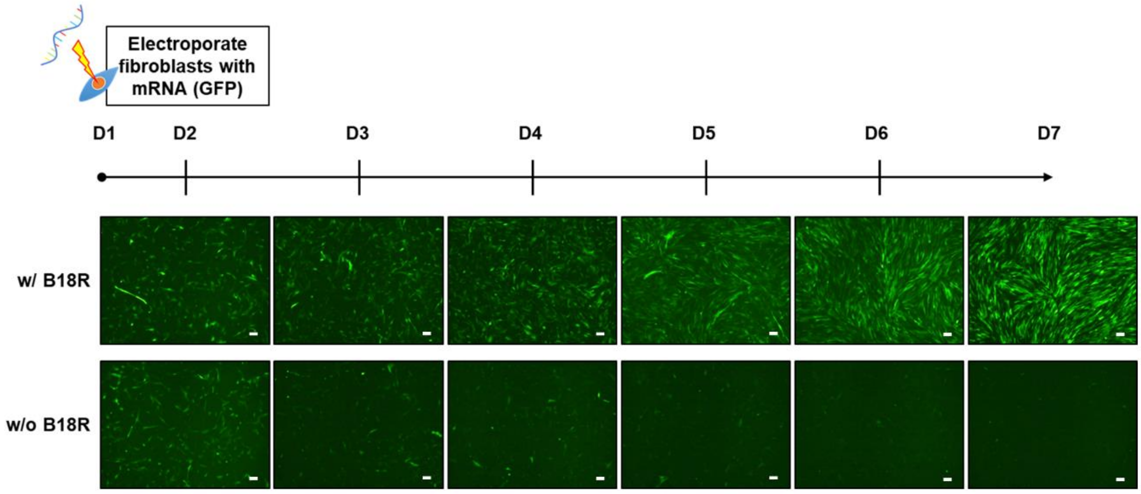

Supplemental Figure S1. Withdrawal of B18R protein diminishes GFP expression induced by synthetic mRNAs. Withdrawal of B18R protein from culture condition diminished the synthetic mRNA-based GFP expression in transfected cells.

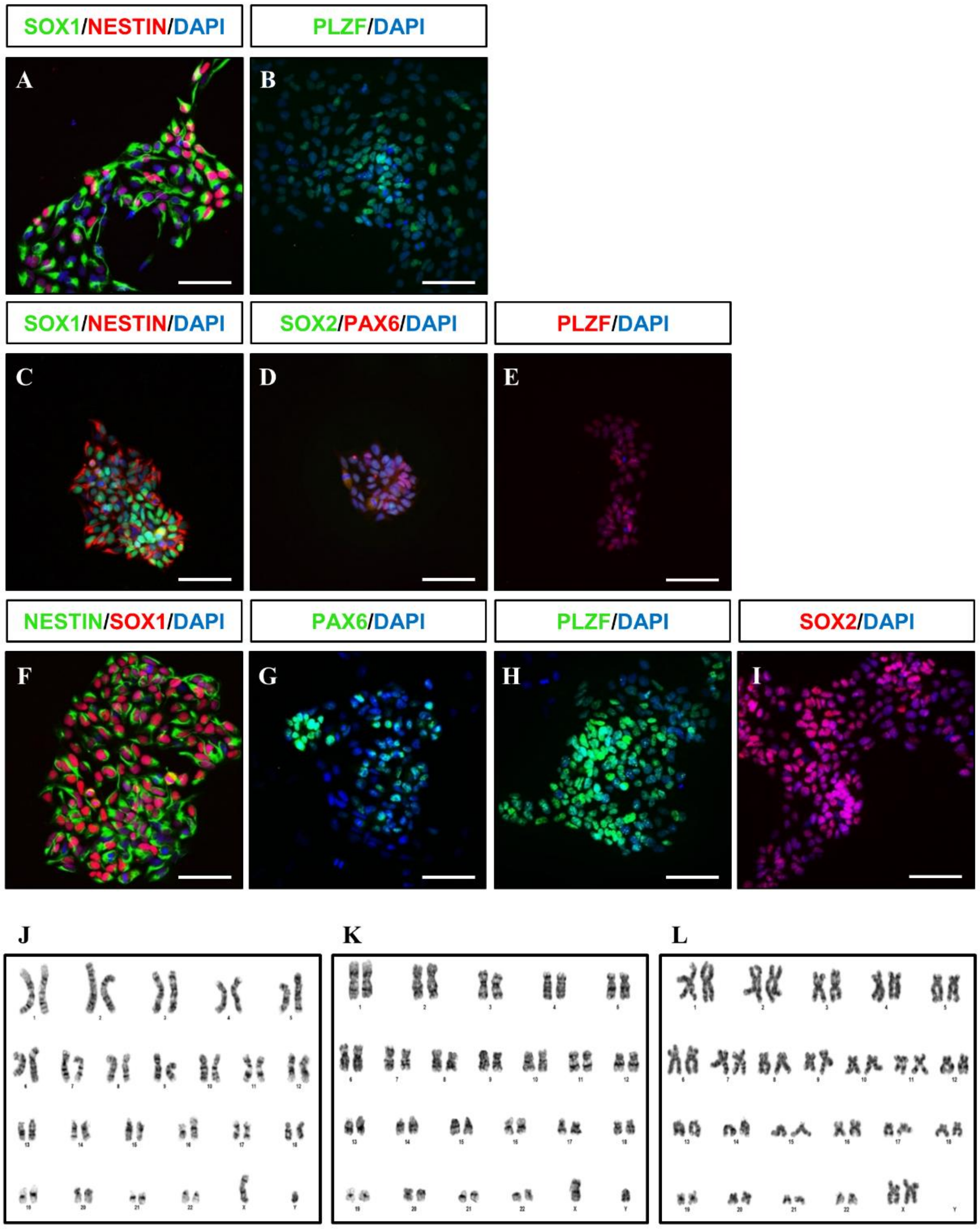

Supplemental Figure S2. Characterization of iNSCs derived from healthy donors. (**A, B**) NSC marker expression of iNSCs from HUCs, derived from male, 31-year-old. (**C-E**) NSC marker expression of iNSCs from HUCs, derived from male, 38-year-old. (**F-I**) NSC marker expression of iNSCs from HUCs, derived from female, 36-year-old. (**J**) Karyotyping of iNSCs from HUCs, derived from male, 31-year-old. (**K**) Karyotyping of iNSCs from HUCs, derived from male, 38-year-old. (**L**) Karyotyping of iNSCs from HUCs, derived from female, 36-year-old. Scale bars, 200 μm.

A

Sample No. *1* *2*

Match Analysis

**Locus**	**Reference Database Profile**		**Sample Profile**		**Shared alleles #**
	**Database : Urine**		**Sample Name : NSC**		
**D5S818**	*8*	*9*	*8*	*9*	*2*
**D13S317**	*9*	*13*	*9*	*13*	*2*
**D7S820**	*11*	*12*	*11*	*12*	*2*
**D16S539**	*11*		*11*		*1*
**vWA**	*16*	*17*	*16*	*17*	*2*
**TH01**	*7*	*9*	*7*	*9*	*2*
**TPOX**	*9*	*11*	*9*	*11*	*2*
**CSF1PO**	*10*	*12*	*10*	*12*	*2*
**AMEL**	*X*	*Y*	*X*	*Y*	*2*
**D3S1358**	*16*		*16*		*1*
**D21S11**	*29*	*30*	*29*	*30*	*2*
**D18S51**	*15*	*18*	*15*	*18*	*2*
**D8S1179**	*12*	*13*	*12*	*13*	*2*
**FGA**	*21*	*21.2*	*21*	*21.2*	*2*
**D2S1338**	*24*	*25*	*24*	*25*	*2*
**D19S433**	*14.2*	*15*	*14.2*	*15*	*2*
**Penta D**	*9*	*12*	*9*	*12*	*2*
**Penta E**	*16*	*17*	*16*	*17*	*2*
Number of shared alleles					*34*
Total number of alleles in the reference database profile					*34*
% match					*100.0%*
**Result interpretation**					**Related**

B

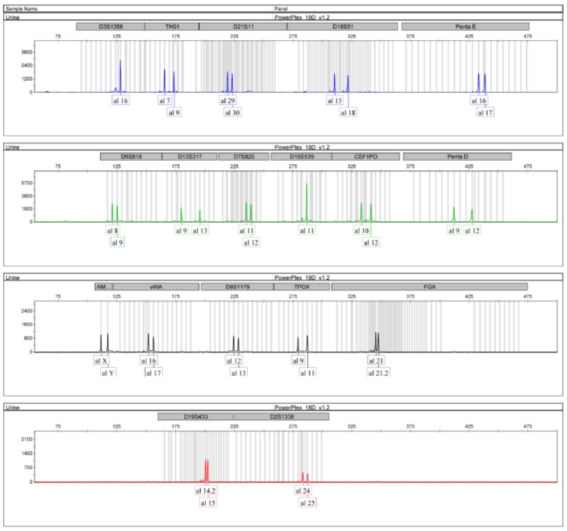

C

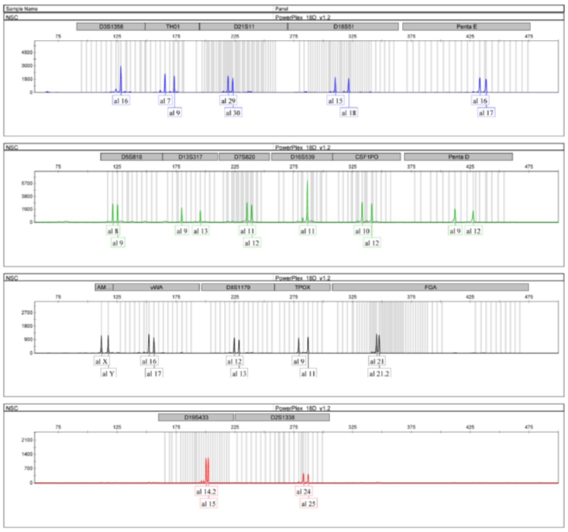

D

Sample No. *1* *2*

Match Analysis

Locus	Reference Database Profile<br>Database : Urine-gang		Sample Profile<br>Sample Name : NSC-gang		Shared alleles #
**D5S818**	10	11	10	11	2
**D13S317**	11	12	11	12	2
**D7S820**	8	12	8	12	2
**D16S539**	9	10	9	10	2
**vWA**	18	19	18	19	2
**TH01**	9		9		1
**TPOX**	8		8		1
**CSF1PO**	12		12		1
**AMEL**	X	Y	X	Y	2
**D3S1358**	16	17	16	17	2
**D21S11**	30	32.2	30	32.2	2
**D18S51**	13	16	13	16	2
**D8S1179**	15	16	15	16	2
**FGA**	20	22	20	22	2
**D2S1338**	18	24	18	24	2
**D19S433**	13	14	13	14	2
**Penta D**	11	12	11	12	2
**Penta E**	11	12	11	12	2
Number of shared alleles					33
Total number of alleles in the reference database profile					33
% match					100.0%
**Result interpretation**					**Related**

E

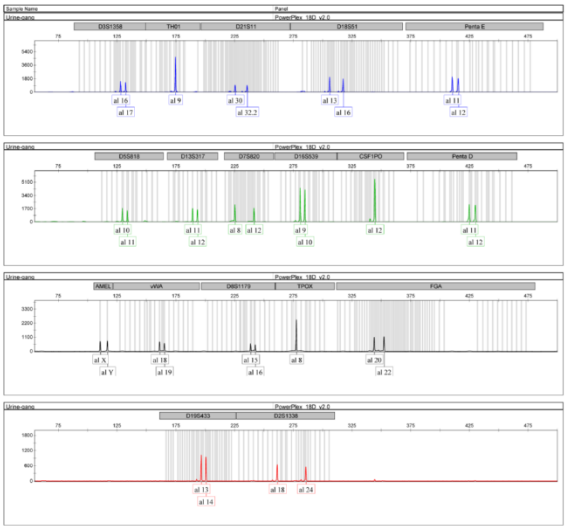

F

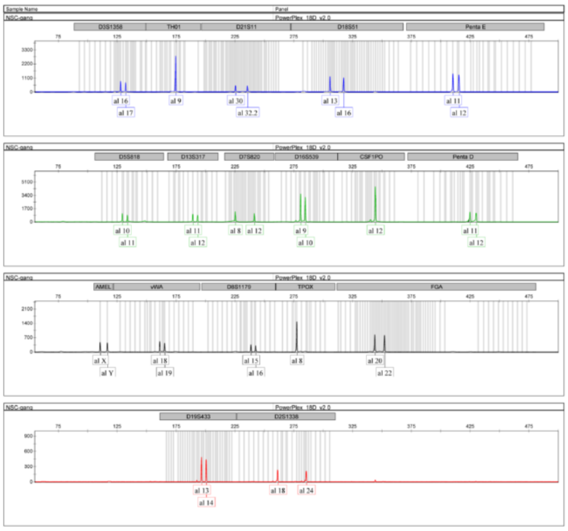

G

Sample No. *1* *2*

Match Analysis

Locus	Reference Database Profile		Sample Profile		Shared alleles #
	Database : Urine no.1 M38		Sample Name : iNSC no.1 M38		
**D5S818**	*9*	*13*	*9*	*13*	*2*
**D13S317**	*8*	*11*	*8*	*11*	*2*
**D7S820**	*10*	*11*	*10*	*11*	*2*
**D16S539**	*9*		*9*		*1*
**vWA**	*17*		*17*		*1*
**TH01**	*9*		*9*		*1*
**TPOX**	*9*	*11*	*9*	*11*	*2*
**CSF1PO**	*10*		*10*		*1*
**AMEL**	*X*	*Y*	*X*	*Y*	*2*
**D3S1358**	*15*	*17*	*15*	*17*	*2*
**D21S11**	*29*	*31*	*29*	*31*	*2*
**D18S51**	*14*	*18*	*14*	*18*	*2*
**D8S1179**	*13*		*13*		*1*
**FGA**	*23*		*23*		*1*
**D2S1338**	*17*	*23*	*17*	*23*	*2*
**D19S433**	*14*	*16.2*	*14*	*16.2*	*2*
**Penta D**	*11*		*11*		*1*
**Penta E**	*10*	*12*	*10*	*12*	*2*
Number of shared alleles					*29*
Total number of alleles in the reference database profile					*29*
% match					*100.0%*
**Result interpretation**					**Related**

H

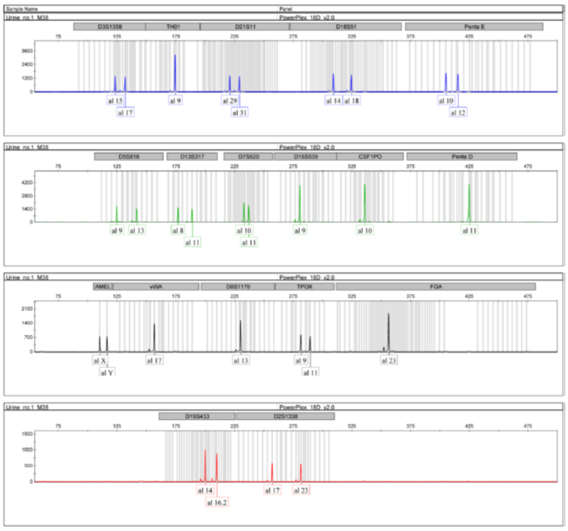

I

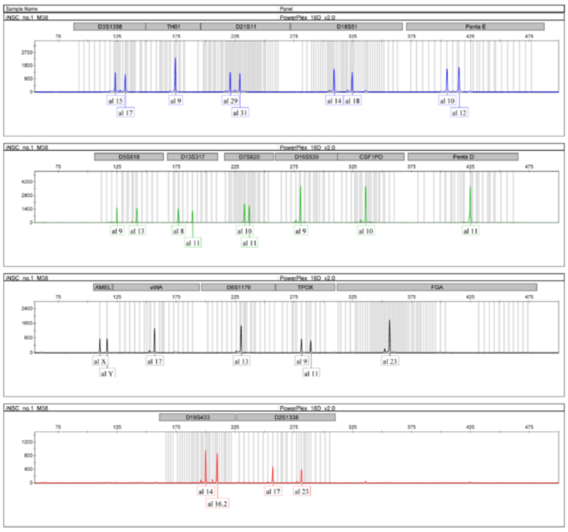

J

Sample No. *1* *2*

Match Analysis

**Locus**	**Reference Database Profile** Database : Urine-EK		**Sample Profile** Sample Name : iNSC-EK		**Shared alleles #**
**D5S818**	*9*	*12*	*9*	*12*	*2*
**D13S317**	*8*	*13*	*8*	*13*	*2*
**D7S820**	*8*	*13*	*8*	*13*	*2*
**D16S539**	*10*		*10*		*1*
**vWA**	*17*		*17*		*1*
**TH01**	*6*	*7*	*6*	*7*	*2*
**TPOX**	*8*		*8*		*1*
**CSF1PO**	*10*	*12*	*10*	*12*	*2*
**AMEL**	*X*		*X*		*1*
**D3S1358**	*16*	*17*	*16*	*17*	*2*
**D21S11**	*29*	*30*	*29*	*30*	*2*
**D18S51**	*16*		*16*		*1*
**D8S1179**	*10*	*11*	*10*	*11*	*2*
**FGA**	*22.2*	*24*	*22.2*	*24*	*2*
**D2S1338**	*19*	*21*	*19*	*21*	*2*
**D19S433**	*12*	*14*	*12*	*14*	*2*
**Penta D**	*9*		*9*		*1*
**Penta E**	*14*	*20*	*14*	*20*	*2*
Number of shared alleles					*30*
Total number of alleles in the reference database profile					*30*
% match					*100.0%*
**Result interpretation**					**Related**

K

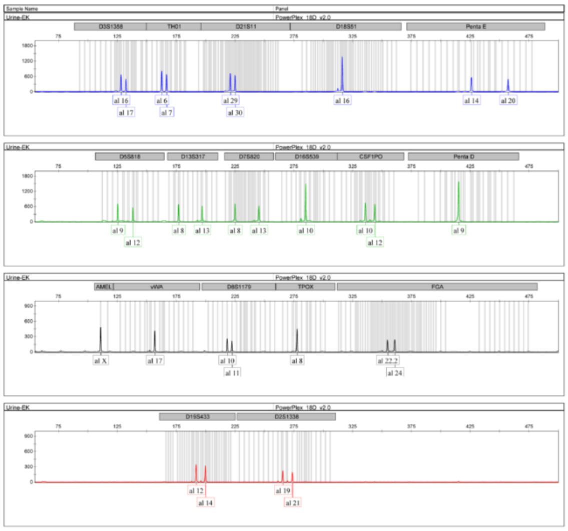

L

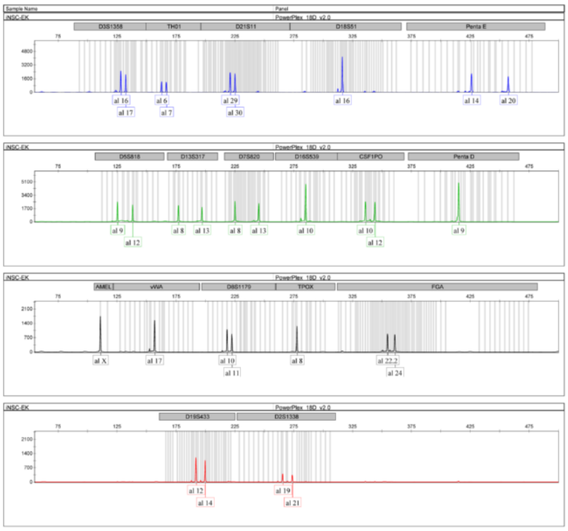

Supplemental Figure S3. STR analysis of iNSCs derived from healthy donors. (**A-C**) STR DNA profiling analysis of parental HUCs and iNSCs, derived from male, 32-year-old. (**B**) and (**C**) are peak data (electropherogram) for parental HUCs and iNSCs, respectively. (**D-F**) STR DNA profiling analysis of parental HUCs and iNSCs, derived from male, 31-year-old. (**E**) and (F) are peak data (electropherogram) for parental

HUCs and iNSCs, respectively. (**G-I**) STR DNA profiling analysis of parental HUCs and iNSCs, derived from male, 38-year-old. (**H**) and (**I**) are peak data (electropherogram) for parental HUCs and iNSCs, respectively. (**J-L**) STR DNA profiling analysis of parental HUCs and iNSCs, derived from female, 36-year-old. (**K**) and (**L**) are peak data (electropherogram) for parental HUCs and iNSCs, respectively.

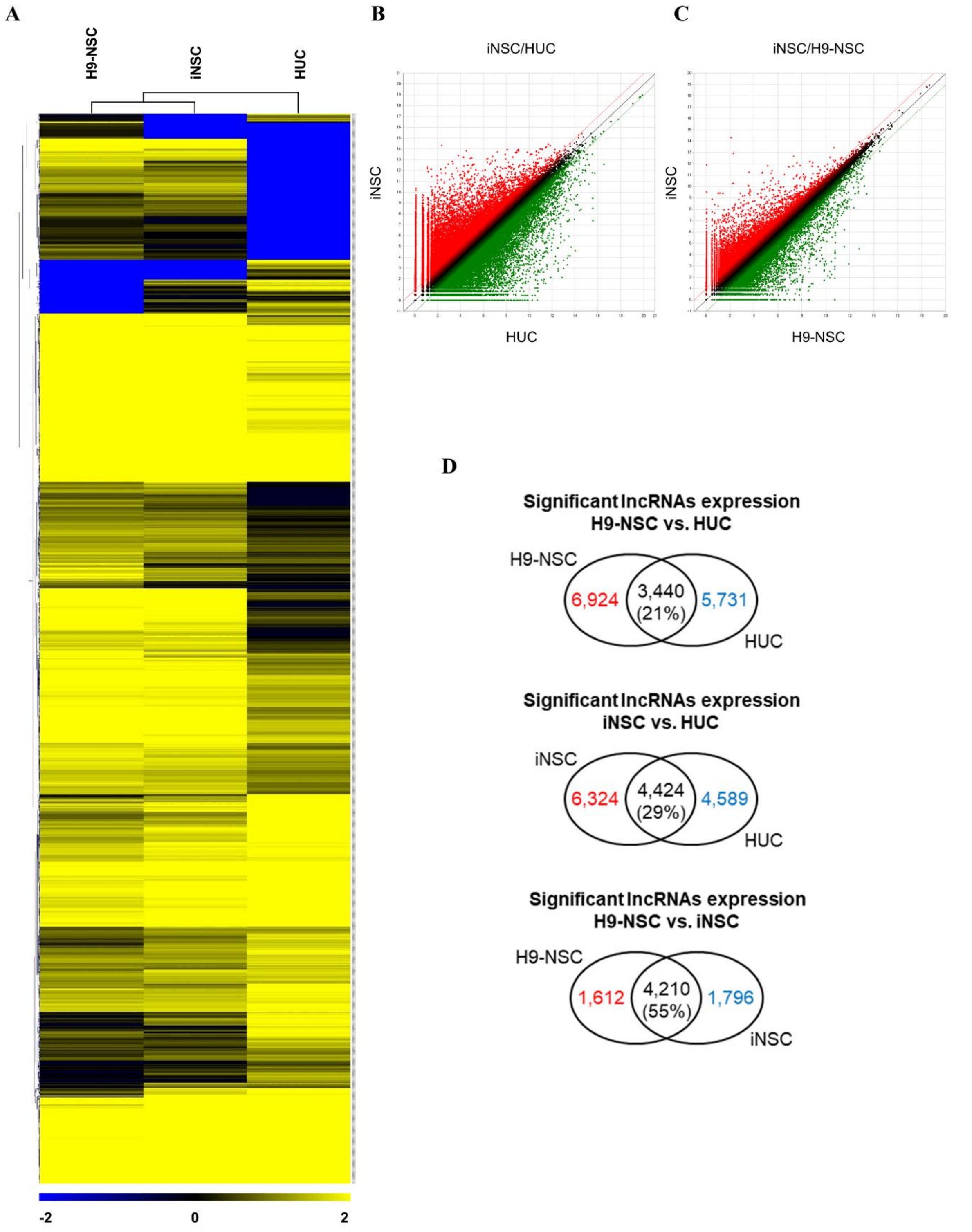

Supplemental Figure S4. RNA sequencing analysis of the lncRNA expression profile of iNSCs. (**A**) Hierarchical clustering analysis of lncRNA expression in H9-NSCs, iNSCs, and HUCs. 12,655 lncRNAs are selected with significant p-value ($p < 0.05$) and fold change ($FC > 2$). (**B, C**) Scatter plot analysis of lncRNAs in iNSC vs. H9-NSCs or HUCs. (**D**) Venn diagrams show significant lncRNAs expressions of H9-NSCs vs. HUCs, iNSCs vs.

HUCs and H9-NSCs vs. iNSCs.

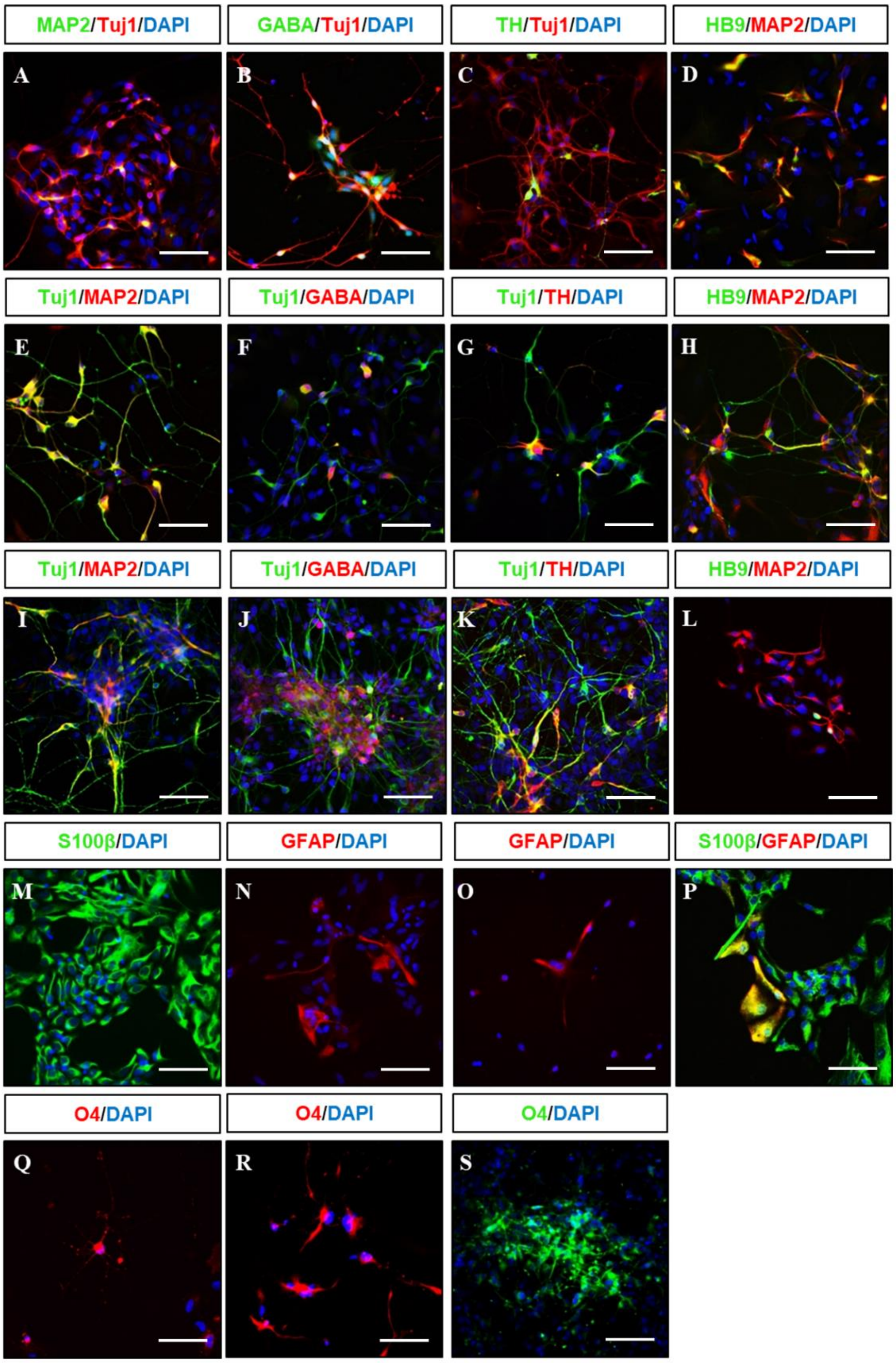

Supplemental Figure S5. Differentiation potential of iNSCs derived from healthy donors. (**A-D**) Neuronal markers expression in iNSC-derived neurons (male, 31-year-old). (**E-H**) Neuronal markers expression in iNSC-

derived neurons (male, 38-year-old). (**I-L**) Neuronal markers expression in iNSC-derived neurons (female, 36-year-old). (**M, N**) Astroglial markers expression in iNSC-derived astroglia (male, 31-year-old). (**O**) Astroglial markers expression in iNSC-derived astroglia (male, 38-year-old). (**P**) Astroglial markers expression in iNSC-derived astroglia (female, 36-year-old). (**Q**) Oligodendroglial markers expression in iNSC-derived oligodendroglia (male, 31-year-old). (**R**) Oligodendroglial markers expression in iNSC-derived oligodendroglia (male, 38-year-old). (**S**) Oligodendroglial markers expression in iNSC-derived oligodendroglia (female, 36-year-old). Scale bars, 200 μm.

Supplemental Table S1. Top 20 up-regulated lncRNAs in iNSCs vs. HUCs.

Top 20 up-regulated lncRNA	lncRNA	Fold change iNSC vs HUC	P-value	Fold change H9-NSC vs HUC	P-value	Chromosome	Start	End	Strand	Proximal gene
1	NONHSAT196622.1	2331.712	0.0000001	1722.544	0.0006476	chr3	181414728	181442487	-	SOX2
2	NONHSAT093477.2	2165.152	0.0000153	1608.971	0.0006533	chr3	181420249	181442508	-	SOX2
3	NONHSAT093478.2	2153.799	0.0000144	1600.403	0.0005219	chr3	181421057	181442486	-	SOX2
4	NONHSAT126980.2	1221.705	0.0000595	1223.597	0.0090088	chr8	65161144	65180341	-	BHLHE22
5	NONHSAT096619.2	1055.934	0.0000384	1185.950	0.0052212	chr4	62117904	62136756	-	ADGRL3
6	NONHSAT058624.2	1001.122	0.0000207	1072.586	0.0000030	chr18	22723490	22778173	-	ZNF521
7	NONHSAT093475.2	818.929	0.0000016	619.977	0.0006395	chr3	181373889	181442508	-	SOX2
8	NONHSAT093476.2	818.929	0.0000016	619.977	0.0006395	chr3	181373889	181442508	-	SOX2
9	NONHSAT123010.2	802.123	0.0010965	1409.944	0.0004956	chr7	121621100	121626826	+	AASS
10	NONHSAT058625.2	772.082	0.0002000	826.394	0.0001181	chr18	22723492	22786873	-	ZNF521
11	NONHSAT178277.1	772.082	0.0002000	826.394	0.0001181	chr18	22723464	22786862	-	ZNF521
12	NONHSAT171697.1	752.393	0.0000179	811.866	0.0038714	chr15	65611231	65655846	-	IGDCC3
13	NONHSAT194978.1	751.779	0.0000183	613.630	0.0008442	chr3	181373888	181442508	+	SOX2
14	NONHSAT058629.2	750.851	0.0007248	872.322	0.0005249	chr18	22784364	22907721	-	ZNF521
15	NONHSAT177503.1	713.078	0.0000104	710.971	0.0042507	chr18	22723487	22797009	+	ZNF521
16	NONHSAT178276.1	682.942	0.0002460	731.430	0.0000116	chr18	22704821	22786859	-	ZNF521
17	NONHSAT162161.1	665.913	0.0005899	757.660	0.0011770	chr12	48385363	48394796	+	COL2A1
18	NONHSAT018342.2	653.464	0.0009344	665.019	0.0074090	chr11	19510892	19524078	-	NAV2
19	NONHSAT006903.2	628.184	0.0000397	441.382	0.0040440	chr1	156641665	156644887	-	NES
20	NONHSAT185419.1	626.346	0.0013783	2108.576	0.0038614	chr2	118061042	118088288	-	DDX18

Supplemental Table S2. . Top 20 down-regulated lncRNAs in iNSCs vs. HUCs.

Top 20 down-regulated lncRNA	lncRNA	Fold change iNSC vs HUC	P-value	Fold change H9-NSC vs HUC	P-value	Chromosome	Start	End	Strand	Proximal gene
1	NONHSAT055470.2	0.0005738	0.0000588	0.0007373	0.0000589	chr17	68101708	68129530	+	KCNJ16
2	NONHSAT090923.2	0.0009164	0.0008068	0.0022107	0.0008098	chr3	101960385	101996532	+	ZPLD1
3	NONHSAT055474.2	0.0009257	0.0001268	0.0018739	0.0001271	chr17	68114411	68135927	+	KCNJ16
4	NONHSAT055460.2	0.0009340	0.0000031	0.0015596	0.0000031	chr17	68101554	68135929	+	KCNJ16
5	NONHSAT055467.2	0.0009342	0.0000035	0.0015597	0.0000035	chr17	68101583	68136002	+	KCNJ16
6	NONHSAT055472.2	0.0009346	0.0000031	0.0015605	0.0000030	chr17	68101563	68133796	+	KCNJ16
7	NONHSAT055465.2	0.0009414	0.0000068	0.0015718	0.0000068	chr17	68101975	68133796	+	KCNJ16
8	NONHSAT073857.2	0.0010438	0.0007494	0.0006061	0.0007487	chr2	113977668	113979984	-	PAX8
9	NONHSAT037288.2	0.0010560	0.0029590	0.0026546	0.0029686	chr14	62117407	62134243	+	HIF1A
10	NONHSAT037289.2	0.0010560	0.0029590	0.0026546	0.0029686	chr14	62117407	62134184	+	HIF1A
11	NONHSAT168547.1	0.0010560	0.0029590	0.0026546	0.0029686	chr14	62117407	62134243	+	HIF1A
12	NONHSAT055457.2	0.0010876	0.0030405	0.0008403	0.0030389	chr17	68096045	68101496	-	KCNJ16
13	NONHSAT055458.2	0.0010885	0.0031414	0.0008410	0.0031398	chr17	68096101	68101496	-	KCNJ16
14	NONHSAT082204.2	0.0011137	0.0003142	0.0014980	0.0003145	chr21	39630270	39726085	+	KCNJ15
15	NONHSAT055464.2	0.0011367	0.0000015	0.0009753	0.0000016	chr17	68101570	68125844	+	KCNJ16
16	NONHSAT055469.2	0.0011388	0.0000075	0.0009771	0.0000076	chr17	68101631	68125840	+	KCNJ16
17	NONHSAT055463.2	0.0011477	0.0000019	0.0009847	0.0000020	chr17	68101567	68125543	+	KCNJ16
18	NONHSAT055468.2	0.0011496	0.0000075	0.0009863	0.0000076	chr17	68101631	68125551	+	KCNJ16
19	NONHSAT175520.1	0.0011508	0.0000165	0.0009874	0.0000166	chr17	68101738	68125790	+	KCNJ16
20	NONHSAT073859.2	0.0012108	0.0003032	0.0006729	0.0003028	chr2	113979568	113983258	+	PAX8
